# Niemann–Pick disease type C in Palestine: genotype and phenotype of sixteen patients and report of a novel mutation in the *NPC1* gene

**DOI:** 10.1186/s12920-021-01072-0

**Published:** 2021-09-17

**Authors:** Imad Dweikat, Othman Thaher, Abdulrahman Abosleem, Almotazbellah Zeer, Ameer Abo Mokh

**Affiliations:** 1 Metabolic Department, Faculty of Medicine, Arab American University, P.O. Box 240, Jenin, West Bank Palestine; 2 Pediatric Department, Faculty of Medicine, Al-Quds University, Abu-Dies, West Bank Palestine

**Keywords:** NPC, Vertical supranuclear gaze palsy, Hepatosplenomegaly, Founder mutation

## Abstract

**Background:**

Niemann–Pick disease type C (NPC) is an autosomal recessive, neurodegenerative disease caused by mutations in either the *NPC1* or *NPC2* genes. Mutations in these genes are associated with abnormal endosomal–lysosomal trafficking, resulting in the accumulation of tissue-specific lipids in lysosomes.

**Methods:**

We described sixteen patients with NPC diagnosed between the age of 1 month and 30 years at two tertiary care centers in Palestine. The clinical phenotype, brain magnetic resonance imaging (MRI), and molecular genetic analysis data were reviewed.

**Results:**

The diagnosis was confirmed by molecular analysis in all patients. Fourteen out of sixteen patients were homozygous for the NPC1 p.G992W variant. Among them, most were categorized as having the late-infantile neurological form of disease onset. They predominantly manifested with early-onset visceral manifestations in the form of hepatosplenomegaly and prolonged neonatal jaundice, and late-onset neuropsychiatric manifestations in the form of vertical supranuclear gaze palsy (VSGP), ataxia, cognitive impairment and seizures. Brain MRI in 6 patients was normal in 5 or consistent with cerebellar hemisphere atrophy in 1 of them. Two other mutations were identified in the NPC1 gene, of which p.V845Cfs*24 was novel.

**Conclusions:**

Our results revealed phenotypic heterogeneity of NPC even within the same genotype, and add to the increasingly recognized evidence that cholestatic jaundice and hepatosplenomegaly during infancy, should alert the physician for the possibility of NPC. We reported a novel mutation in the *NPC1* gene further expanding its genotype.

## Background

Niemann–Pick disease type c (NPC) disease is a rare, pan-ethnic autosomal recessive, neurovisceral lysosomal storage disease with an incidence of 1 in 100,000–120,000 live births. It stems from inherited deficiencies of lysosomal proteins involved in intracellular lipid-trafficking and characterized by accumulation of unesterified cholesterol and glycolipids in the lysosomes and late endosomes. It is caused by mutations in either the *NPC1* gene (95% of cases) or the *NPC2* gene (5% of cases) [1]. The clinical presentation of NPC is extremely heterogeneous, with the age of onset ranging from the perinatal period until well into adult age. Clinical manifestations can be categorized into visceral and neuropsychiatric symptoms [2]. Visceral symptoms include hepatosplenomegaly, isolated splenomegaly and prolonged cholestatic jaundice in neonates and young infants. Neuropsychiatric features include vertical supranuclear gaze palsy (VSGP), cerebellar ataxia, dysarthria, dysphagia, progressive dementia, cataplexy, seizures and dystonia [1, 2]. In a large national study from the USA, the average age of deaths due to NPC disease is 16.2 years, and one-half of them died before the age of 12.5 years [3]. Early diagnosis is of vital importance so that therapy with miglustat can be initiated as soon as neurological symptoms appear in order to slow the progression of the disease or stabilize the patient’s neurological deterioration [4, 5].

The age of onset of neurological symptoms determines the speed of progression and the prognosis of the disease [1]. Generally, patients with early onset deteriorate faster and die sooner. Categorization of patients by age at onset of neurological manifestations has led to the definition of early-infantile (at age 3 months to < 2 years), late-infantile (at age 2 to < 6 years), juvenile (6–15 years) and adolescent/adult (> 15 years)-onset disease forms [1, 5].

Herein, we report the clinical, radiological and molecular findings of 16 patients at two tertiary care centers in the West Bank, Palestine. The phenotype was that of early-onset visceral symptoms followed later by development of neurological or neuropsychiatric symptoms. We also described their genotype and reported a novel variant, p.V845Cfs*24 “frameshift: insertion mutation”. It was identified as a novel homozygous 1-bp insertion in exon 17 of the *NPC1* gene further expanding the genotype of the disease. This is, to our knowledge, the only study to describe phenotype and genotype of Niemann–Pick disease type C in Palestine.

## Methods

This is a retrospective analysis of sixteen patients with NPC, diagnosed between the age of 1 month and 30 years on the basis of the clinical manifestations, brain imaging and molecular genetic analysis. The majority of patients were referred for evaluation because they had features suggestive of a non-specific neurometabolic disorder, especially in the context of a previously affected family member, while the others were referred in the early infantile period because of a previous confirmed diagnosis of NPC in one of the family members. The clinical suspicion was based on visceral, neurological and psychiatric manifestations (Table 1). These data were obtained from the patients’ medical records, in addition to information obtained from the parents during follow-up clinic visits. The patients were divided into four groups according to the age of onset of neurological manifestations: Early-infantile, late-infantile, juvenile and adolescent/adult-onset forms.

**Table 1:** Clinical phenotype and genotype of NPC patients

Patient no	Gender, ID and age	Consanguinity	Visceral and neurological manifestations, age of onset and clinical outcome	Brain MRI	Mutation
1	F, AA^a^ 8 years	1st cousins	Hepatosplenomegaly at 6 months. Developmental delay at 2 years. Aggressive behavior, temper tantrums and sleep disturbances at 3 years. VSGP, ataxia, gelastic cataplexy, frequent falls, urinary and fecal incontinence at 5 years	Normal	p.G992W (G > T)
2	F, JA^a^ 4 years	1st cousins	Neonatal cholestatic jaundice. Hepatosplenomegaly at 2 weeks. Isolated splenomegaly at 1½ years. Developmental delay at 2 years	Not performed	
3	M, MM^e^ 20 years	1st cousins	Neonatal cholestatic jaundice. Hepatosplenomegaly at 1 week. Ascites at 3 months. Low platelets at 5 years. VSGP at 11 years. Cognitive impairment, hyperactivity, poor concentration and aggressive behavior at 13 years. Ataxia at 15 years. Frequent falls, swallowing problems, speech problems, tonic–clonic seizure, absence seizure and gelastic cataplexy at 16 years	Normal	
4	F, EA^e^ 4 years	1st cousins once removed	Neonatal cholestatic jaundice. Hepatosplenomegaly at 2 weeks	Not performed	
5	F, DA^e^ 2 years	1st cousins	Neonatal cholestatic jaundice. Hepatosplenomegaly at 1 month	Not performed	
6	M, AS^b^ 2 years	1st cousins	Neonatal cholestatic jaundice. Hepatosplenomegaly and central hypotonia at 3 months. Mild speech delay, normal cognition, behavior and gait at 2 years	Not performed	
7	M, MS^b^ 6 years	1st cousins	Neonatal cholestatic jaundice. Hepatosplenomegaly, ascites and thrombocytopenia at 3 months. Central hypotonia and swallowing problems at 1 year. Sleep disturbance at 3 years. Ataxia at 4 years	Not performed	
8	M, YS^c^ 9 years	1st cousins	Splenomegaly at 3 years. Developmental delay at 2 years. Gelastic cataplexy, cognitive impairment, hyperactivity and poor attention at 4 years. Urinary and fecal incontinence at 5 years. VSGP, speech problem and swallowing problems at 6 years. Ataxia, frequent falls, spasticity and tonic–clonic seizure at 6.5 years	Increased signal intensity on T2-weighted images in the cerebral white matter	
9	M, AS^c^ 12 years	1st cousins	Hepatosplenomegaly at 3 years. VSGP, frequent falls and developmental delay at 2½ years. Ataxia at 3½ years. Speech problem and swallowing problems at 4 years. Spasticity at 4½ years. Gelastic cataplexy and cognitive impairment at 5 years. Absence seizure at 10½ years	Not performed	
10	M, MS^c^ 7 years^f^	1st cousins	Hepatosplenomegaly at 2 years. VSGP, spasticity and inability to walk at 4 years. Speech and swallowing problems, gelastic cataplexy, urinary and fecal incontinence at 5 years. Tonic-clonic seizure and cognitive impairment at 6 years	Not performed	
11	F, SY^d^ 14 years	1st cousins	Splenomegaly and VSGP at 5 years. Ataxia at 6 years	Normal	
12	F, SA^a^ 13 years	1st cousins	Splenomegaly, VSGP, ataxia, speech and swallowing problems, absence seizure, cognitive impairment and sleep disturbance at 5 years. Gelastic cataplexy at 7 years	Normal	
13	F, RS^b^ 7½	1st cousins	Hepatosplenomegaly at 1 year. Frequent falls at 3 years. VSGP, ataxia, swallowing and speech problems, gelastic cataplexy, cognitive impairment, poor school performance, aggressive behavior and sleep disturbances at 4 years	Not performed	
14	F, GY^d^ 26 years^f^	1st cousins	Ataxia at 15 years. VSGP and cognitive impairment and abnormal behavior at 20 years. Spasticity at 21 years. Speech problem and swallowing problems at 22 years. Frequent falls at 23 years. Urinary incontinence at 24 years	Not performed	
15	F, IZ 31 years	2nd cousins	Ataxia at 19 years. VSGP and spasticity at 21 years. Splenomegaly, Speech and swallowing problems at 24 years. Urinary and fecal incontinence at 27 years. Cognitive impairment and abnormal behavior at 28 years	Atrophic changes in the cerebellar hemisphere	A927V (C > T)
16	M, KH 2 years	1st cousins	Cholestatic jaundice. Hepatosplenomegaly at 3 months. Hypotonia and motor delay at 1 year. Thrombocytopenia at 1 year	Normal	p.V845Cfs*24 (Novel)

^a^Patients (#1, #2, #12) belong to the same family

^b^Patients (#6, #7, #13) belong to the same family

^c^Patients (# 8, #9, #10) belong to the same family

^d^Patients (#11, #14) belong to the same families

^e^Patients (#3, #4, #5): Each belong to a different family

^f^The patient was dead at the time of conducting the study

### Molecular analysis

The definite diagnosis of NPC was confirmed by molecular genetic analysis in all patients. Blood samples from the patients were used to isolate DNA by standard methods. Coding sequences and flanking regions of the *NPC1* and the *NPC2* genes were amplified with polymerase chain reaction (PCR), purified and submitted to direct DNA sequencing. Informed consent for molecular analyses was obtained from all patients.

## Results

We reported sixteen patients (9 females and 7 males) of NPC disease aged from 1 month to 30 years belonging to 9 families. All patients were offspring of consanguineous marriages. Fourteen patients were alive at the time of the study, 2 patients died at the ages of 7 years and 26 years respectively. Fourteen out of sixteen patients were homozygous for the p.G992W variant. Of these, visceral manifestations were present in 13 patients (92%). 8 patients (57%) present in the first year with cholestatic jaundice and hepatosplenomegaly. Two patients had ascites and thrombocytopenia (Table 1). Of note, 2 patients (#4, # 5) did not develop neurological symptoms at age 4 years and 2 years respectively.

The age of onset and the speed of progression of neurological symptoms in patients with the p.G992W was heterogenous. Central hypotonia was identified in the infantile period in 2 patients (#6, #7). 8 patients (57%) had the late-infantile form (#1, #2, #8, #9, #10, #11, #12, #13) and 1 patient had the juvenile form (#3). The most common symptoms in the two age groups were developmental delay, VSGP, gelastic and absence seizure, ataxia, frequent falls, swallowing and speech problems, cognitive impairment, and abnormal behavior. One patient (#10) had severe phenotype leading to death at age 7 years. The affected children had increasing motor and intellectual disabilities through late childhood and adolescence, especially VSGP, ataxia, dysphagia and progressive deterioration of academic and cognitive skills, ultimately leading to abstinence from school. One patient (14) manifested during adolescent/adult period with progressive neurological symptoms including ataxia, VSGP, swallowing and speech deterioration, urinary incontinence, spasticity and cognitive impairment leading to death at age 26 years (Table 1). Patients in this age group were characterized by prominent neuropsychiatric disturbances with insidious onset and slow progression. Retrospectively, other visceral and neurological symptoms were also retrieved. Despite this late presentation, there was a substantial gap between the age of presentation and age when the diagnosis of NPC was confirmed.

Brain MRI was only performed for six patients with the p.G992W variant and revealed abnormalities in two patients including increased signal intensity on T2-weighted MRI images in the cerebral white matter in one patient, and cerebellar hemisphere atrophy was noted in the other patient. In the other four patients, no abnormalities in cerebral white matter, basal ganglia, thalami and cerebellar hemisphere.

The diagnosis was confirmed by molecular genetic analysis (Tables 1, 2). All patients have homozygous mutations in the *NPC1* gene, with no reported mutations in the *NPC2* gene. The fourteen patients homozygous for the pathogenic mutation p.G992W were from 7 families, thirteen of them were from a single governorate but they belonged to different unrelated families. This missense mutation (Table 2) was first detected by Greer et al. [6] in the Nova Scotian genetic isolates. One patient (#15) was homozygous for the mutation p.Ala927Val which is a missense transition-type pathogenic variant that converts alanine 927 to valine [7]. She presented in the adulthood period with ataxia, VSGP, spasticity, speech and swallowing problems, frequent falls, urinary and fecal incontinence, cognitive impairment and splenomegaly. One patient (#16) was homozygous for the novel mutation p.V845Cfs*24 “frameshift: insertion mutation”. This mutation was identified as a novel homozygous 1-bp insertion in exon 17 out of 25 of the *NPC1* gene chr18:21120484 insC resulting in premature termination after 24 codons (Table 2). The patient with this mutation, currently aged 2 years, had prolonged neonatal jaundice, hepatosplenomegaly, ascites and thrombocytopenia, followed at age of 1 year by hypotonia and motor delay. Other missense mutations in the same exon have been previously reported as pathogenic. Family Segregation analysis confirmed that both parents and the other two siblings were heterozygous for the mutation. The findings on molecular genetic analysis in this patient also correlated with the clinical phenotype which included prolonged neonatal jaundice, hepatosplenomegaly, ascites and hypotonia.

**Table 2:** Description of the reported mutations in the *NPC1* gene and the pathogenicity prediction scores

Number of patients	Mutation type	Exon	Zygosity	Pathogenicity prediction scores
14 patients	c.2974 G > T p.G992W “point mutation” missense	20	Homozygous	It was reported as pathogenic variant in Nova Scotia patients (reference 15). It is a point mutation that causes a G 2974 → T transversion which converts glycine 992 to tryptophan. It was found in 8 clones from two patients and was not observed in cDNAs derived from unaffected individuals. This single base change abolishes a Bsu361 restriction-enzyme recognition site It was also reported as pathogenic in 2 Arab Muslim families in Israel (reference 7)
1 patient	Chr18:21120484 insC c.2531dpG p.V845Cfs*24 Novel “frameshift: insertion mutation”	17	Homozygous	To our knowledge, it has not been reported previously, neither as a pathogenic nor as a benign one. We believe it is likely pathogenic because it introduces a premature stop codon at codon 869 which creates a premature translational stop signal. These changes may result in nonsense mediated mRNA decay as predicted by Mutation Taster (http://www.mutationtaster.org/) This variant was not observed in 1000 genomes, ExAC, and gnomAD databases, indicating it is not a common benign variant in these populations The variant was found to segregate with the disease phenotype in the family. Loss-of-function variants in NPC1 gene are known to be pathogenic Based on the standards and guidelines of American College of Medical Genetics and Genomics (ACMGG), we assessed this variant as being pathogenic
1 patient	c.2780 C > T A927V “point mutation” missense	18	Homozygous	It was previously reported as pathogenic in an Arab Muslim family in Israel (reference 7)

Substrate reduction therapy with miglustat was administered only to four patients because the cost of the therapy was beyond the financial capacities of most families. The duration of therapy ranged from 4 months to 1 year making it difficult to give a conclusion of its effect on neurological status.

## Discussion

NPC disease is a rare panethnic autosomal recessive neurodegenerative disease. The broad clinical spectrum ranges from devastating neonatal illness to progressive neurodegenerative disease that occurs in childhood and adults. The rate of progression and life span show considerable variation. Disease severity and the age at which neurological symptoms begin largely correlate with the prognosis of the disease [1]. Wide heterogeneous and complex presentation of the disease are a great challenge for making the diagnosis in clinical practice. Understanding the natural history of the disease is necessary to make early diagnosis and assessment of affected patients. This is important in improving the knowledge and raising the awareness of healthcare providers about diagnosis and management of the disease.

In our study, we have presented sixteen patients from different families with NPC disease. Clinical manifestations, brain MRI and molecular genetic analysis were reported. Hepatosplenomegaly, isolated splenomegaly and prolonged neonatal jaundice were the most frequently observed visceral symptoms. These findings were consistent with the findings in the literature that the visceral manifestations are strong clinical indications of the disease [2, 8]. Early consideration of NPC disease in the differential diagnosis of these manifestations is important to prevent intensive and unnecessary investigations.

The late-infantile form with onset of neurological symptoms between 2 and 6 years was the most commonly observed phenotype in our patients with the p.G992W mutation. The most common neuropsychiatric symptoms were VSGP, ataxia, swallowing and speech problems, cognitive impairment, seizure and gelastic cataplexy (Table 1) but neurological symptoms were identified in all age groups from neonatal period to adulthood. In accordance with the literature, delay in developmental motor milestones, hypotonia and language/speech delay were the earliest manifestations in early childhood. Cerebellar ataxia, dysarthria, dysphagia, progressive dementia, gelastic cataplexy, seizures, dystonia and VSGP became more frequent with increasing age [1, 5]. Of these manifestations, VSGP and gelastic cataplexy are the strongest neurological indicators for NPC [8]. Coexistence of VSGP and hepatosplenomegaly detected clinically should greatly raise the suspicion of the disease [9]. Similarly, in general, psychiatric disorders, mainly cognitive impairment and dementia, were more common in adolescent and adult NPC cases [2].

More than 40% of patients with all forms of NPC present in the first year of life with cholestatic jaundice and visceromegaly [10, 11]. In the late-infantile form of our series, 2 patients had hepatosplenomegaly (AS, MS) and 3 patients had isolated splenomegaly (YS, SY). Splenomegaly was also observed in the patient with the p.Ala927Val variant at the age 24 years [3, 11, 12]. Similarly, the onset of neurological symptoms in our series were insidious and appeared after a varying period of normal or slightly delayed development [4, 11].

Brain imaging is one amongst the non-invasive modalities that can be useful in offering possible supplementary explanations of the neurological symptoms in NPC patients [13]. The characteristic cerebellar and basal ganglia pathology detected through neuroimaging may explain ataxia, speech problems, VSGP and cognitive impairment [14]. Another abnormal change that can be detected is cerebral atrophy with signal enhancement of the white matter [12, 15, 16]. Despite all these findings, no one has shown high specificity for the NPC diagnosis. In our study, brain MRI was performed in seven patients and showed abnormalities only in two patients. This could be partially explained by the fact that brain MRI was performed at a younger age, before the onset of neurological symptoms and by the small number of the patients who underwent brain MRI. Unfortunately, two patients were dead at the time of conducting the study, and performing brain MRI was denied by the parents of the other patients.

NPC disease is caused by genetic mutations in either *NPC1* or *NPC2* genes that lead to impairment of intracellular trafficking of cholesterol. It is highly advisable to do genetic testing, since nowadays it is the highly preferred modality to confirm or disprove diagnosis. The three different mutations found in the molecular genetic analysis of our patients were p.Ala927Val, p.V845Cfs*24 and the most common one, p.G992W.

p.G992W mutation is a known pathogenic mutation of the *NPC1* gene. It was previously known as Niemann Pick disease type D (NPD) and is the typical mutation of the Acadian Nova-Scotian patients, where it is subject to a founder effect [6, 17]. Also, it has been reported sporadically but rarely in patients of other origins [18]. It was also found in two unrelated Muslim Arab families originating from the northern parts of Israel [7]. One patient presented with hepatosplenomegaly and the other presented with hepatosplenomegaly and mental retardation [7]. This mutation accounts for the majority of the clinical phenotype of NPC in our series consisting of neonatal and infantile onset of prolonged jaundice and hepatosplenomegaly and neuropsychiatric symptoms mainly VSGP, ataxia, speech and swallowing problems, cognitive impairment, frequent falls and gelastic epilepsy during childhood and adolescence. In accordance with previous reports, our patients showed inter- and Intrafamilial phenotypic variability [7]. The patient with p.Ala927Val mutation also had similar phenotype consisting of hepatosplenomegaly during infantile period, followed by ataxia, VSGP, spasticity, speech and swallowing problems, and cognitive impairment during adolescence. This mutation has been previously detected in a Muslim family living in Israel; the patient had mild phenotype consisting of mild splenomegaly and ataxia at age 20 years [7]. It was also reported in two patients from Czech Republic typically manifesting with the adolescent/adult-onset form of NPC [10].

The main phenotype of the patient with the novel mutation p.V845Cfs*24 in our series is typical of the early infantile neurological form, and rather expected in a child homozygous for a null mutation.

Founder mutations are often responsible for the high prevalence of rare genetic disorders in specific populations and awareness of ethnic background allows for targeted screening. Identification of these mutations in various ethnic groups is an extremely important step to screen and diagnose the disease and to provide genetic counseling especially among Palestinian people because of high rates of consanguineous marriages. In addition, it gives an excellent opportunity for preimplantation and prenatal genetic diagnosis of the disease.

NPC disease is one of the genetic diseases that can be treated with miglustat, a substrate reduction therapy approved by European Medicines Agency (EMA). Also, it was approved by other countries such as Canada, Australia and Japan. In USA, it can be used for treatment of NPC only as an off label medication. Treatment can be considered at an early onset of neurological manifestations [19]. In other countries, miglustat has been used for treatment of the neurological manifestations of the disease. However, in the West Bank, it is not available at an affordable price which limits its use among NPC patients.

## Conclusions

NPC may be an under-recognized inherited neurovisceral disorder because of the heterogeneity of the clinical presentation. It should be suspected in patients presenting with splenomegaly/hepatosplenomegaly and prolonged cholestatic jaundice in neonates or young infants, especially with the later development of neurological and psychiatric manifestations. History of previous diagnosis of NPC disease in some family members contributed significantly to an earlier diagnosis of the disease in other family members. We reported a novel pathogenic mutation in the *NPC1* gene further expanding the genotype of the disease.

## Data Availability

The datasets generated and/or analysed during the current study are available in the ClinVar (http://www.ncbi.nlm.nih.gov/clinvar/) repository, and can be found under the accession number SCV001653519.1/RCV000020230.8, and SCV001653518.1/RCV001449976.1 for NM_000271.5(NPC1):c.2974G > T (p.Gly992Trp), and NM_000271.5(NPC1):c.2531dup (p.Val845fs), respectively.

## Abbreviations

NPC: Niemann–Pick disease type C
VSGP: Vertical supranuclear gaze palsy
PCR: Polymerase chain reaction
MRI: Magnetic resonance imaging
